# Supplementary material for: Roles of miR-640 and Zinc Finger Protein 91 (ZFP91) in Angiopoietin-1-Induced In Vitro Angiogenesis
Source: Cells. 2020 Jul 2;9(7):1602. doi: 10.3390/cells9071602 (PMC7408170; doi:10.3390/cells9071602)
Supplement: Supplementary file 1 [file cells-09-01602-s001.pdf]

Supplementary Materials

Table S1. Primers used for quantitative real-time PCR experiments.

Gene	Sequence (5’⇒3’)	Accession #	Expected size (bp)
β-ACTIN	F: AGAAAATCTGGCACCACACC<br>R: GGGGTGTTGAAGGTCTCAAA	NM_001101	126
Ang-1	F: GCAGAGAGATGCTCCACACG<br>R: TATCTGGGCCATCTCCGACT	NM_001146	145
BAFF	F: GGGAGCAGTCACGCCTTAC<br>R: GATCGGACAGAGGGGCTTT	NM_0006573	103
CCL19	F: TACATCGTGAGGAACTTCCACT<br>R: CTGGATGATGCGTTCTACCCA	NM_006265	132
GAPDH	F: AAGAAGGTGGTGAAGCAGGCG<br>R: ACCAGGAAATGAGCTTGACAA	NM_023964	166
VEGF	F: CTACCTCCACCATGCCAAGT<br>R: CACACAGGATGGCTTGAAGA	NM_001025366	281
ZFP91	F: CTCGCTATTTGCAGCACCAC<br>R: GCCCGAGCACAATATTCACA	NM_053023	165
Pre-miR-640	F: TGGGCAAGTTCCTGAAGATCAGA<br>R: AGAGGCAGGTTCCTGGATCAT	MIMAT0003310	
Hsa-miR-640	GATCCAGGAACCTGCCTCT	MIMAT0003310	
U6	ACTAAAATTGGAACGATACAGAGA	NR_004394	
5S	ATACCGGGTGCTGTAGGCTT	D14867	

	Predicted consequential pairing of target region (top) and miRNA (bottom)	Site type	Context++ score
Position 125-131 of ZFP91 3' UTR<br>hsa-miR-640	5' ...CUGCUCUUUCCAUUGUGGAUCAC...<br>||||||| 3' UCUCCGUCCAAGGACCUAGUA	7mer-m8	-0.02
Position 349-355 of ZFP91 3' UTR<br>hsa-miR-640	5' ...AACUCUGAUGUGCUCUGGAUCAG...<br>||||||| 3' UCUCCGUCCAAGGACCUAGUA	7mer-m8	-0.04
Position 3096-3103 of ZFP91 3' UTR<br>hsa-miR-640	5' ...AUUGAACAAAUGUAAUGGAUCAA...<br>||||||| 3' UCUCCGUCCAAGGACCUAGUA	8mer	-0.41

	Context++ score percentile	Weighted context++ score	Conserved branch length	P_{CT}
Position 125-131 of ZFP91 3' UTR<br>hsa-miR-640	16	-0.02	0.054	N/A
Position 349-355 of ZFP91 3' UTR<br>hsa-miR-640	44	-0.04	0.054	N/A
Position 3096-3103 of ZFP91 3' UTR<br>hsa-miR-640	98	-0.41	0.054	N/A

Figure S1. Predicted miR-640 binding regions located in human ZFP91 3′UTR (derived from TargetScan Release 7.1).

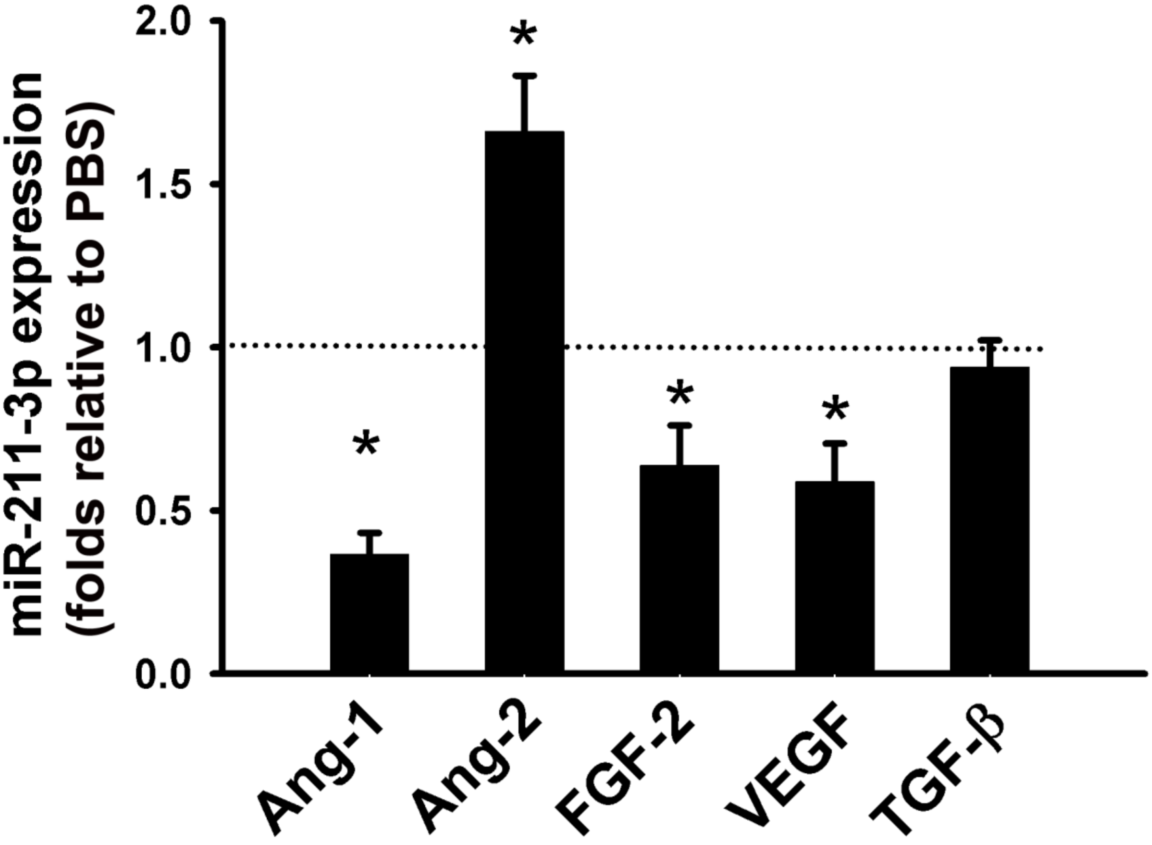

Figure S2. Regulation of of miR-211-3p expression in ECs by angiogenesis factors. Mature miR-211-3p expression measured in HUVECs exposed to PBS, Ang-2 (300ng/ml), FGF-2 (10ug/ml), VEGF (40ng/ml), or TGF-β (2ng/ml) for 24 h. Values are means± SEM and are expressed as folds relative to PBS. *P < 0.05, compared with PBS.

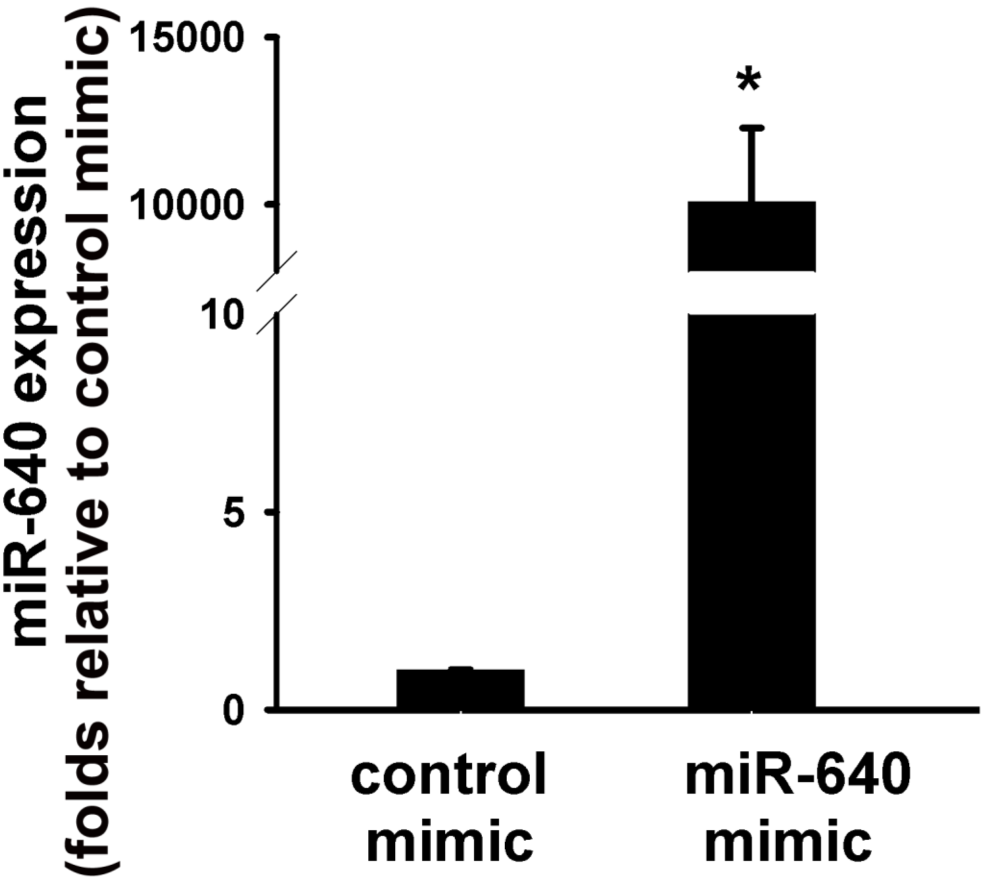

Figure S3. Transfection efficiency of miR-640 mimic in HUVECs. miR-640 levels in HUVECs transfected with control or miR640 mimic. Mean ±SEM, *$p<0.05$, compared to control mimic.

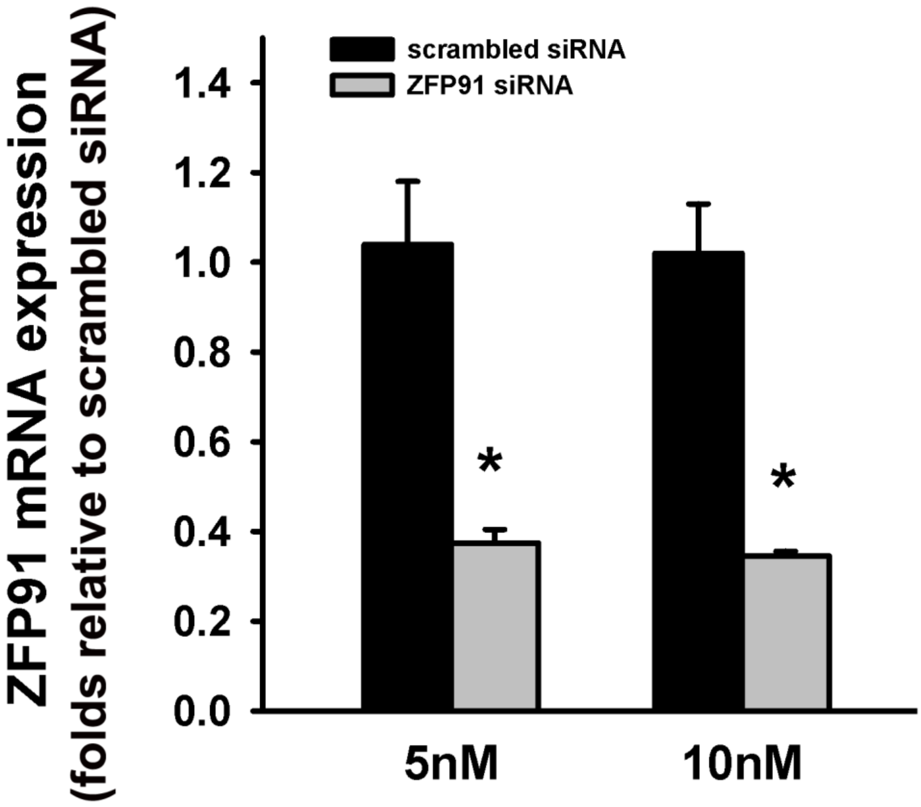

Figure S4. Verification of ZFP91 knockdown by siRNA oligos. ZFP91 mRNA expression measured in HUVECs transfected with scrambled or ZFP91 siRNA oligos. Values were measured 48 h post-transfection. *P<0.05, compared to scrambled siRNA.
